# Susceptibility to SARS-CoV-2 of Cell Lines and Substrates Commonly Used to Diagnose and Isolate Influenza and Other Viruses

**DOI:** 10.3201/eid2705.210023

**Published:** 2021-05

**Authors:** Li Wang, Xiaoyu Fan, Gaston Bonenfant, Dan Cui, Jaber Hossain, Nannan Jiang, Gloria Larson, Michael Currier, Jimma Liddell, Malania Wilson, Azaibi Tamin, Jennifer Harcourt, Jessica Ciomperlik-Patton, Hong Pang, Naomi Dybdahl-Sissoko, Ray Campagnoli, Pei-Yong Shi, John Barnes, Natalie J. Thornburg, David E. Wentworth, Bin Zhou

**Affiliations:** Centers for Disease Control and Prevention, Atlanta, Georgia, USA (L. Wang, X. Fan, J. Hossain, M. Currier, M. Wilson, A. Tamin, J. Harcourt, J. Ciomperlik-Patton, H. Pang, N. Dybdahl-Sissoko, R. Campagnoli, J. Barnes, N.J. Thornburg, D.E. Wentworth, B. Zhou);; Oak Ridge Institute for Science and Education, Oak Ridge, Tennessee, USA (G. Bonenfant, N. Jiang, G. Larson);; Battelle Memorial Institute, Atlanta, Georgia, USA (D. Cui, J. Liddell);; University of Texas Medical Branch, Galveston, Texas, USA (P.-Y. Shi)

**Keywords:** angiotensin-converting enzyme 2, ACE2, cell lines, coronavirus disease, COVID-19, influenza, MDCK, respiratory infections, SARS-CoV-2, spike protein substitution, severe acute respiratory syndrome coronavirus, viruses, zoonoses

## Abstract

Co-infection with severe acute respiratory syndrome coronavirus 2 (SARS-CoV-2) and other viruses has been reported. We evaluated cell lines commonly used to isolate viruses and diagnose related diseases for their susceptibility to SARS-CoV-2. Although multiple kidney cell lines from monkeys were susceptible to SARS-CoV-2, we found many cell types derived from humans, dogs, minks, cats, mice, and chicken were not. We analyzed MDCK cells, which are most commonly used for surveillance and study of influenza viruses, and found that they were not susceptible to SARS-CoV-2. The low expression level of the angiotensin converting enzyme 2 receptor and lower receptor affinity to SARS-CoV-2 spike, which could be overcome by overexpression of canine angiotensin converting enzyme 2 in trans, strengthened the cellular barrier to productive infection. Moreover, a D614G mutation in the spike protein did not appear to affect SARS-CoV-2 cell tropism. Our findings should help avert inadvertent propagation of SARS-CoV-2 from diagnostic cell lines.

Coronavirus disease (COVID-19) has resulted in >70 million laboratory-confirmed cases and >1.6 million deaths in <1 year since the first case was confirmed. Co-infection with severe acute respiratory syndrome coronavirus 2 (SARS-CoV-2) and other viruses, such as influenza virus, has been reported (*1*–*4*). Because cases of COVID-19 continue to climb sharply, more coinfections are expected, especially in the current and future influenza seasons.

Isolating and propagating viruses from clinical specimens in cell cultures or embryonated chicken eggs is widely used to identify multiple viruses and produce vaccines, mostly under Biosafety Level 2 containment. Currently, SARS-CoV-2 must be isolated and propagated under Biosafety Level 3 containment because of its risk to laboratorians and the general public. Therefore, if any of these cell lines or eggs support productive replication of SARS-CoV-2, then a validated procedure should be implemented to rule out the presence of SARS-CoV-2 in the specimens before their inoculation. However, adding a diagnostic step specific to SARS-CoV-2 in many circumstances is impractical or substantially increases the cost and labor required.

We conducted this study to determine whether cell lines and eggs commonly used to isolate and propagate influenza viruses, poliovirus, and other human viruses can support productive replication of SARS-CoV-2. If a substrate is confirmed to be insusceptible to SARS-CoV-2, modifying procedures to diagnose and isolate susceptible viruses in that substrate may be unnecessary. Although we repeated all results under the same or slightly different conditions, some of our results were further confirmed using multiple assay methods on divergent SARS-CoV-2 strains and in cell lines from different sources. Our study provides additional information on the risk of inadvertently propagating SARS-CoV-2 in cell lines and substrates when isolating, identifying, propagating, or producing vaccines for other viruses. 

## Materials and Methods

### Viruses

We used 3 virus stocks for our investigation. The SARS-CoV-2/USA-WA1/2020 (USA-WA1) viral strain was isolated from the specimen of the first confirmed case in the United States (*5*). SARS-CoV-2/Massachusetts/VPT1/2020 (MA/VPT1) was isolated in Vero E6 cells from a nasopharyngeal specimen collected in April 2020. The recombinant fluorescent reporter virus icSARS-CoV-2-mNG was generated as described elsewhere (*6*). We sequenced the spike genes of all working stocks. Although USA-WA1 and MA/VPT1 did not have mutations or variations (at the 20% cutoff level), icSARS-CoV-2-mNG acquired a 5-residue insertion at the furin cleavage site resulting in a sequence change from “PRRARS” to “PRRNIGERARS” in most (≈70%) of the viral population. Although furin cleavage site mutations were reported to decrease entry and infection efficiency to various degrees in lung epithelial cells (*7*–*9*), because ≈30% of the population in our working stock contains the intact furin cleavage site, we still used it in the qualitative assessment of SARS-CoV-2 entry of various cell lines.

### Cells

We obtained MDCK-Atlanta, MDCK-London, and MDCK-SIAT1 cells from International Reagent Resources (https://www.internationalreagentresource.org) and MDCK-hCK cells from the University of Wisconsin–Madison (https://www.wisc.edu). We obtained MDCK-NBL2, Vero E6, CV-1, A549, Crandell-Rees Feline Kidney (CRFK) cells, Mv1Lu, RD, Hep-2c, HeLa, and L20B cells from American Type Culture Collection (https://www.atcc.org); these cells were maintained at Division of Scientific Resources, National Center for Emerging and Zoonotic Infectious Diseases, Centers for Disease Control and Prevention (Atlanta, GA, USA). We obtained chicken embryo fibroblasts from Charles River Laboratories (https://www.criver.com). We obtained an additional 25 cell lines (Table 1) from Quidel Corporation (https://www.quidel.com); these lines were preseeded in 24-well plates, except for CRFK and rhesus monkey kidney cells, which were obtained in T-75 flasks and seeded into 24-well plates in the laboratory 1 day before infection.

**Table 1:** Overview of commercial cell lines used in study of susceptibility to SARS-CoV-2 of cell lines and substrates used to diagnose and isolate influenza and other viruses*

Cell line	Organism	Tissue	Type/ morphology	Virus susceptibility profile†	SARS-CoV-1 susceptible (references)	SARS-CoV-2 susceptible
Vero	African green monkey	Kidney	Epithelial	AdV, coxsackie B, measles, mumps, rotavirus, rubella, influenza	Yes (*32*,*38*)	Yes
Vero 76	African green monkey	Kidney	Epithelial	AdV, coxsackie B, measles, mumps, poliovirus, rotavirus, rubella, West Nile Virus	Yes (*39*)	Yes
BGMK	African green monkey	Kidney	Epithelial	coxsackie B, poliovirus	Yes (*32*)	Yes
CV-1	African green monkey	Kidney	Fibroblast	measles, mumps, rotavirus	Yes (*32*)	No
LLC-MK2	Rhesus macaque	Kidney	Epithelial	enterovirus, myxovirus and poxvirus groups, poliovirus type 1, rhinovirus	Yes (*32*)	Yes
RhMK	Rhesus macaque	Kidney	Epithelial	enteroviruses, influenza, parainfluenza	Yes (*35*)	Yes
A549	Human	Lung	Epithelial	AdV, influenza, measles, mumps, parainfluenza, poliovirus, RSV, rotavirus	No (*32*,*34*,*35*); Yes (*40*)	No
HEL	Human	Lung	Fibroblast	AdV, CMV, echovirus, HSV, poliovirus, rhinovirus	No (*32*,*35*)	No
HeLa	Human	Cervix	Epithelial	AdV, CMV, echovirus, HSV, poliovirus, rhinovirus	No (*32*)	No
HeLa 229	Human	Cervix	Epithelial	AdV, CMV, echovirus, HSV, poliovirus, rhinovirus	Unknown	No
HEp2	Human	Cervix	Epithelial	AdV, coxsackie B, HSV, measles, parainfluenza, poliovirus, RSV	No (*32*)	No
MRC-5	Human	Lung	Fibroblast	AdV, CMV, echovirus, HSV, influenza, mumps, poliovirus, rhinovirus	No (*35*)	No
MRHF	Human	Foreskin	Fibroblast	AdV, CMV, echovirus, HSV, mumps, poliovirus, rhinovirus	Unknown	No
NCI-H292	Human	Lung	Epithelial	AdV, HSV, influenza A, measles virus, RSV, rhinoviruses, vaccinia virus	No (*34*,*37*,*40*)	No
RD	Human	Muscle	Spindle; multinucleated	AdV, echovirus, HSV, poliovirus	No (*32*,*36*)	No
WI-38	Human	Lung	Fibroblast	AdV, CMV, echovirus, HSV, influenza, mumps, poliovirus, rhinovirus, RSV	Unknown	No
McCoy	Mouse	Unknown	Fibroblast	HSV	Unknown	No
MNA	Mouse	Nerve	Neuroblastoma	Rabies	Unknown	No
MDCK	Dog	Kidney	Epithelial	AdV, coxsackie virus, influenza, reoviruses	No (*29*,*32*,*33*,*35*,*37*)	No
CRFK	Cat	Kidney	Epithelial	canine parvovirus, feline calicivirus, feline panleukopenia virus, rabies virus	Yes (*29*)	Yes (limited)
Mv1Lu	American mink	Lung	Epithelial	CMV, influenza	Yes (*35*,*38*)	No
H&V-Mix	CV-1 and MRC-5	Mixture	Mixture	AdV, CMV, echovirus, HSV, influenza, poliovirus type 1, SV40 virus, VZV	Unknown	No
R-Mix	Mv1Lu and A549	Mixture	Mixture	AdV, CMV, HSV, influenza, measles, mumps, poliovirus, RSV, rotavirus	Yes (*35*)	No
R-Mix Too	MDCK and A549	Mixture	Mixture	AdV, HSV, influenza, MPV, measles, mumps, poliovirus, RSV, rotavirus, VZV	Unknown	No
Super E-Mix	BGMK and A549	Mixture	Mixture	AdV, HSV, influenza, measles, mumps, poliovirus, RSV, rotavirus, VZV	Unknown	Yes

*AdV, adenovirus; CMV, cytomegalovirus; HSV, herpes simplex virus; RhMK, rhesus monkey kidney; RSV, respiratory syncytial virus; VZV, varicella zoster virus. †Virus susceptibility profiles listed are as reported by Quidel (https://www.quidel.com) and not verified in this study.

### Virus Infection of Cell Lines

We seeded cells in 6-, 12-, or 24-well plates 1 day before infection or used them directly upon receipt from Quidel. Infection dose for each experiment is specified in the results section or figure legends. In general, inoculum was saved for back titration and the result is shown as 0 hours postinoculation (hpi) in some figures. We then washed cells at 1–2 hpi and collected supernatants or cell lysates daily for up to 3 days for infectious virus titration and up to 5 days hpi for viral RNA quantification. We observed cytopathic effect and fluorescence signals for icSARS-CoV-2-mNG daily.

### Virus Infection of Embryonated Chicken Eggs

We obtained specific pathogen-free embryonated chicken eggs from Charles River Laboratories. We inoculated USA-WA1 into the allantoic cavity of twenty-four 8- to 12-day-old eggs at 10^5^ median tissue culture infectious dose (TCID_50_)/egg and incubated them at 37°C for 3 days. Allantoic fluid was collected from individual eggs separately as E1 samples. We passaged 100 µL of each E1 sample into a corresponding egg and collected 24 E2 samples after 3 days of incubation. We also generated 24 E3 samples from passage of E2 samples in 24 eggs. We titrated all E1, E2, and E3 samples, as well as samples from cell lines, with TCID_50_ assay using VeroE6 cells; viral RNAs were quantified by real-time reverse transcription PCR (rRT-PCR) (*10*). We used synthetic RNA in the rRT-PCR assay to generate the standard curve for absolute quantification.

### Immunoblot Detection and PCR Quantification of Angiotensin-Converting Enzyme 2

Cells were lysed in NP-40 lysis buffer and we determined protein concentrations using a Pierce BCA protein assay kit (https://www.thermofisher.com). We immunoblotted cell lysates and recombinant angiotensin-converting enzyme (ACE) 2 protein control (Sino Biological; https://www.sinobiological.com) for ACE2 and β-actin using 1:500 polyclonal goat anti-human ACE2 AF933 (R&D Systems; https://www.rndsystems.com) and 1:1,000 monoclonal mouse anti-β-Actin AB8226 (Abcam; https://www.abcam.com) primary antibodies followed by Abcam 1:4,000 donkey anti-goat and 1:4,000 goat anti-mouse secondary antibodies (Biorad; https://www.bio-rad-antibodies.com or KPL; https://www.seracare.com). We developed immunoblots using ThermoFisher SuperSignal West Pico PLUS chemiluminescent substrate. Qualitative RT-PCR (qRT-PCR) was used to determine the relative mRNA ACE2 levels in different cell lines. Two sets of primers and probes (Table 2) were used for each cell type targeting identical regions of ACE2 mRNA multiplexed with Applied Biosystems 4310893E eukaryotic 18S rRNA (https://www.thermofisher.com). We used the comparative cycle threshold (ΔΔCt) method to quantify relative ACE2 gene expression. For each cell type and primer/probe set, we normalized ACE2 cycle threshold against 18S rRNA and then standardized to Vero E6.

**Table 2:** Primers and probes used for the quantification of ACE2 mRNA in various cell lines in study of susceptibility to SARS-CoV-2 of cell lines and substrates used to diagnose and isolate influenza and other viruses

Assay identification	Applicable cell lines	Primers/probes*	Sequence, 5′ → 3′
ACE2.FAM.10	Vero E6, A549, CRFK, CV-1	Forward	CCCAGAATCCTTGAGTCAT
		Probe	TACTGATGCAATGGTGAACC
		Reverse	TTGGACAGAAACCAAACATAG
ACE2.FAM.11	Vero E6, CRFK	Forward	GGGTCACAGTATGTTTCATC
		Probe	TATCTCTCGCTTCATCTCCC
		Reverse	GGAGGTGGATGGTCTTTA
ACE2.FAM.12	Vero E6, MDCK-NBL-2, MDCK-SIAT1	Forward	TGGTCTTTGGGAATTTCA
		Probe	TAAAGACCATCCACCTCCAC
		Reverse	GAAATCATGTCACTTTCTGC
ACE2.FAM.13	Vero E6, MDCK-NBL-2, MDCK-SIAT1	Forward	AACATGGAACAGAGATGC
		Probe	CCAAAGACCAGTGGATGAAA
		Reverse	GGAGGTGGATGGTCTTTA
ACE2.FAM.14	Vero E6, Mv1Lu	Forward	CTTCATAGTCTCCTCTCCAATAA
		Probe	CTCTTCATATAATGGCCTCAGC
		Reverse	CTACAATGAGAGGCTCTGG
ACE2.FAM.15	Vero E6, Mv1Lu	Forward	CTCTTCATATAATGGCCTCAG
		Probe	AGACTACAATGAGAGGCTCT
		Reverse	ATGAGCACCATCTACAGT
ACE2.FAM.16	Vero E6, A549, CV-1	Forward	GGGTCACAGTATGTTTCATC
		Probe	TATCTCTCGCTTCATCTCCC
		Reverse	GGAGGTGGATGGTCTTTA

*Probes labeled at the 5′-end with the reporter molecule 6-carboxyfluorescein (FAM), internally with the quencher ZEN, and at the 3′-end with Iowa Black FQ (Integrated DNA Technologies, https://www.idtdna.com).

### Expression of Recombinant ACE2 Proteins and Biolayer Interferometry Assay

We used the ThermoFisher Expi293 Expression system to produce histidine-tagged ACE2 (ectodomain) proteins and purified them using HisTrap FF column (GE Life Sciences, https://www.cytivalifesciences.com) as described elsewhere (*11*). We evaluated affinity between Sino Biologic 40591-V02H SARS-CoV-2 S1 and human ACE2 or canine ACE2 using ForteBio anti–penta-His (HIS1K) biosensors (https://www.sartorius.com) on Octet RED96 at 30°C with a shaking speed at 1,000 RPM. We corrected the data by subtracting reference sample and used 1:2 bivalent binding model with global fit to determine affinity constants.

### Exogenous Expression of ACE2 in MDCK Cells and ACE2 Sequence Alignment

We generated constructs coexpressing full-length human ACE2 (hACE2) or canine ACE2 (cACE2) with mCherry2 protein (CMV promoter-ACE2-IRES-mCherry2) and transfected them into MDCK-SIAT1 cells through electroporation with the Lonza Nucleofector system (https://bioscience.lonza.com) using the manufacturer’s protocol with program A024. We transfected 1.5 × 10^6^ MDCK-SIAT1 cells with 10 µg DNA (pCMV-hACE2-IRES-mCherry2, pCMV-cACE2-IRES-mCherry2, or pCMV-IRES-mCherry2 empty control). One day posttransfection, we inoculated the cells with USA-WA1 or icSARS-CoV-2-mNG. We aligned ACE2 protein sequences for human (GenBank accession no. NP_001358344.1), African green monkey (accession no. AAY57872.1), rhesus macaque (accession no. ACI04564.1), mouse (accession no. NP_001123985.1), dog (accession no. XP_005641049.1), cat (accession no. NP_001034545.1), American mink (accession no. QPL12211), and chicken (accession no. XP_416822.2) using MUSCLE alignment in Geneious Prime software version 2019.2.3 (https://www.geneious.com).

## Results

### Replication of SARS-CoV-2 in a Large Set of Cell Substrates

**Figure 1:**
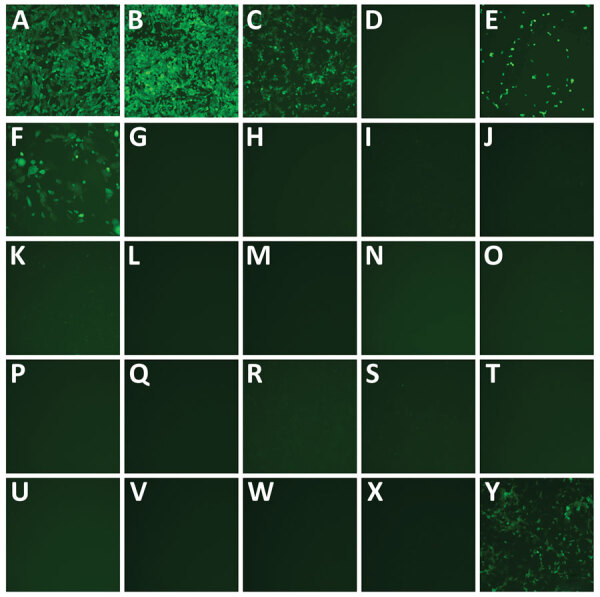
Select commercially sourced cell lines infected by severe acute respiratory syndrome coronavirus 2 (SARS-CoV-2) in study of susceptibility to SARS-CoV-2 of cell lines and substrates used to diagnose and isolate influenza and other viruses. A) Vero; B) Vero 76; C) BGMK; D) CV-1; E) LLC-MK2; F) RhMK; G) A549; H) HEL; I) HeLa; J) Hela 229; K) Hep-2; L) MRC-5; M) MRHF; N) NCI-H292; O) RD; P) WI-38; Q) McCoy; R) MNA; S) MDCK; T) CRFK; U) Mv1Lu; V) H&V-Mix; W) R-Mix; X) R-Mix Too; Y) Super E-Mix. Cell lines were inoculated with the SARS-CoV-2 reporter virus encoding mNeonGreen (icSARS-CoV-2-mNG) and infected cells (green fluorescence). Microscopy images (original magnification ×10) captured 1 day postinfection, but similar results were observed through 5 days postinfection; all mNeonGreen-negative cell lines remained negative.

**Figure 2:**
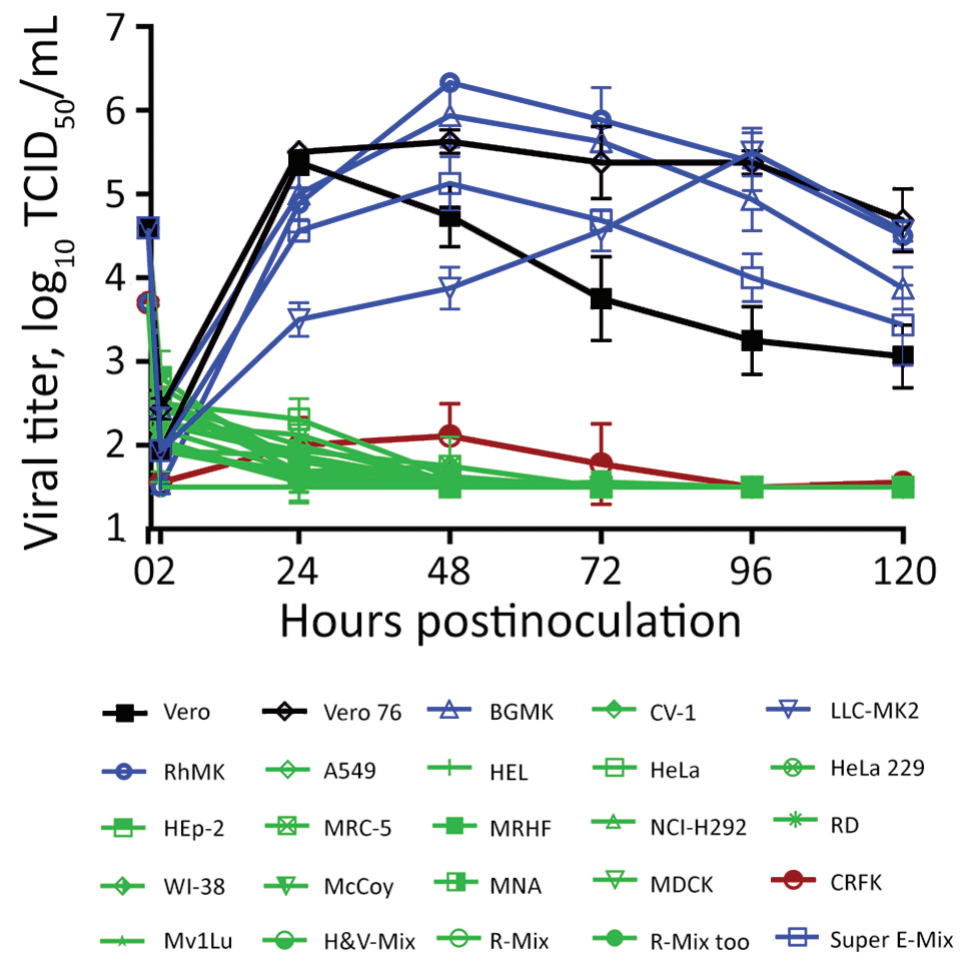
Varied severe acute respiratory syndrome coronavirus 2 (SARS-CoV-2) viral replication kinetics in commercially sourced cell lines in study of susceptibility to SARS-CoV-2 of cell lines and substrates used to diagnose and isolate influenza and other viruses. Data are mean of n = 4 +SD. TCID_50_, median tissue culture infectious dose.

We seeded the 25 cell lines from Quidel in 24-well plates and inoculated with 5 × 10^4^ TCID_50_/well of a fluorescent reporter virus in which the open reading frame 7a gene was replaced by the mNeonGreen gene (icSARS-CoV-2-mNG), allowing successful infection to be visualized by a green fluorescence signal (*6*). Almost all nonhuman primate cell lines were susceptible to icSARS-CoV-2-mNG infection except for CV-1 cells (Figure 1). In contrast, none of the tested human, mouse, mink, dog, or cat cell lines yielded fluorescent cells after infection. The Super-E Mix cells were likely susceptible because this cell culture is a mixture containing BGMK cells, which were found to be susceptible to SARS-CoV-2 (Figure 1). We then inoculated all these cell lines with 5 × 10^4^ TCID_50_/well of the wild type SARS-CoV-2/USA-WA1/2020 (USA-WA1) strain and titrated supernatants collected over 5 days. Consistent with the results from icSARS-CoV-2-mNG infection, all nonhuman primate cell lines except CV-1 cells supported productive virus replication, whereas all other cell lines failed to generate infectious virus (Figure 2). It should be noted that viral titers in CRFK cells increased slightly at 2 days postinfection (dpi) (Figure 2), suggesting that this cell line may support a low level of replication. 

### Replication of SARS-CoV-2 in Influenza Virus Substrates

Laboratories use multiple lineages or derivatives of MDCK cells and embryonated chicken eggs to isolate and propagate different types or subtypes of influenza viruses. Some lineages, such as MDCK-SIAT1 and hCK cells, were genetically modified and cloned from single cells, resulting in altered cell morphology and enhanced susceptibility to some subtypes of influenza viruses compared with susceptibility in their parental MDCK cell lines (*12*,*13*). The different lineages of MDCK cells have altered gene expression profiles and surface glycans and it is unclear whether that would affect their susceptibility to SARS-CoV-2. Therefore, we examined the susceptibility to SARS-CoV-2 in representative lineages of MDCK cells that are widely used in different laboratories, including MDCK-NBL-2, MDCK-Atlanta, MDCK-London, MDCK-SIAT1, and MDCK-hCK.

**Figure 3:**
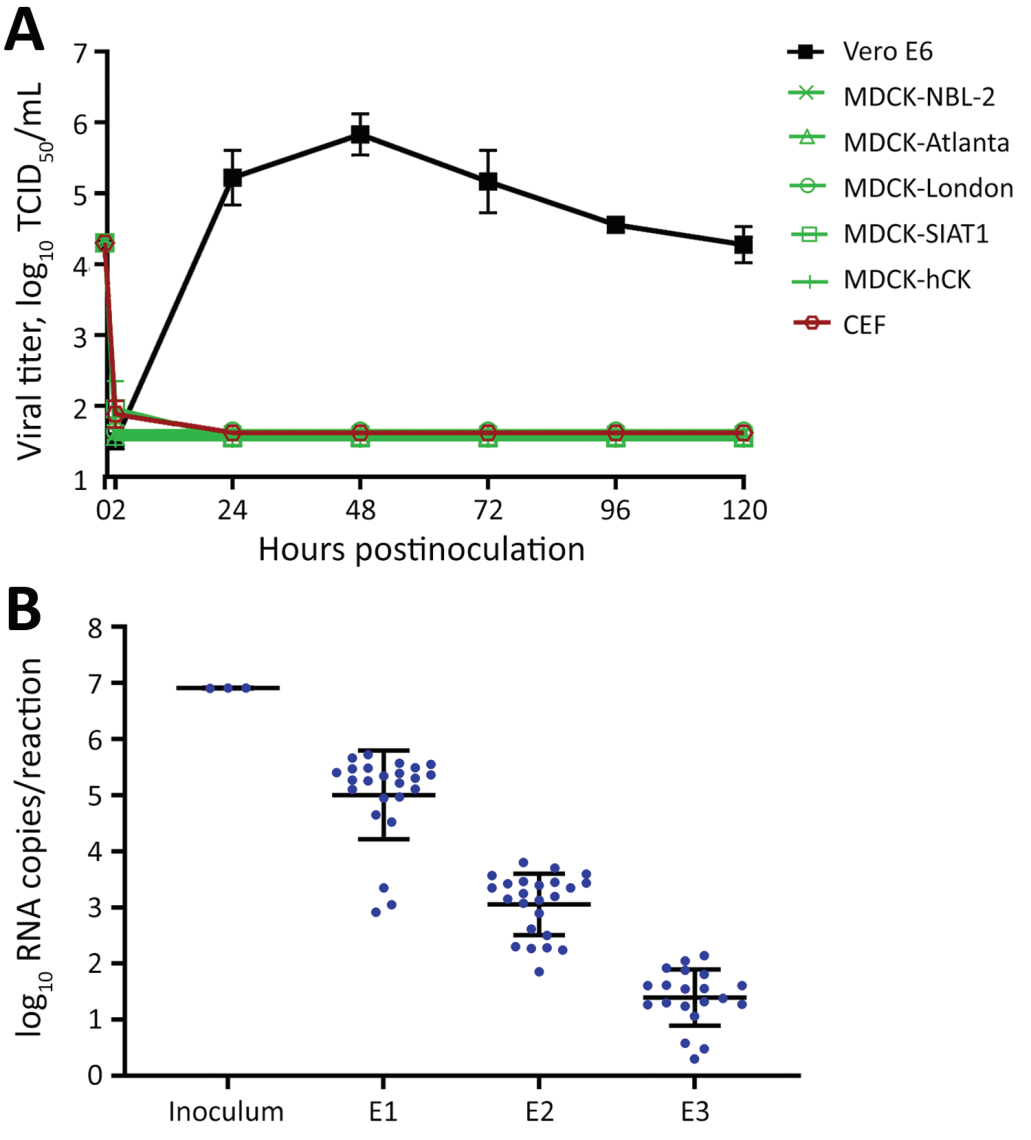
Influenza virus substrates not infected by severe acute respiratory syndrome coronavirus 2 (SARS-CoV-2) in study of susceptibility to SARS-CoV-2 of cell lines and substrates used to diagnose and isolate influenza and other viruses. A) Vero E6, MDCK-NBL-2, MDCK-Atlanta, MDCK-London, MDCK-SIAT1, MDCK-hCK, and chicken embryo fibroblast cells inoculated with USA-WA1 at 5 × 10^4^ TCID_50_/well in 12-well plates (MOI 0.1 to ≈0.3, depending on cell line). B) USA-WA1 total viral RNA levels in allantoic fluid from infected eggs quantified by real-time reverse transcription PCR using a standard curve generated by synthetic RNA. Four eggs with undetectable RNA not plotted for E3. Data are mean of n = 3 +SD (cells) or n = 24 +SD (eggs). TCID_50_, median tissue culture infectious dose.

We inoculated Vero E6 cells as a positive control and various MDCK cell lines with 5 × 10^4^ TCID_50_/well of USA-WA1 and incubated for 1–2 hours at 37°C. We then washed cells to remove the inoculum and influenza virus infection media containing TPCK-trypsin and added bovine serum albumin to mimic the conditions used to isolate influenza viruses. We collected supernatants at the indicated times postinfection and measured viral titers. Vero E6 cells supported robust viral replication and reached peak titer in <2 days (Figure 3, panel A), and infection killed most cells (data not shown). In contrast, none of the 5 MDCK cell lines tested supported SARS-CoV-2 replication. Although residual infectious virus was present in some MDCK supernatant samples at 2 hpi, it was below the limit of detection at 1 dpi and did not cause any cytopathic effect through 5 dpi. We conducted similar experiments with the MDCK cell lines in which the infection media contained fetal bovine serum rather than bovine serum albumin and again SARS-CoV-2 failed to replicate in any of the 5 MDCK cell lines (data not shown but almost identical to Figure 3, panel A).

Embryonated chicken eggs are another common substrate for isolating, propagating, and producing vaccines for influenza viruses. We inoculated 24 eggs each with 10^5^ TCID_50_ of USA-WA1 and blindly passaged the virus in eggs for 3 passages (E1, E2, and E3). Viral titers in the allantoic fluid of E1, E2, and E3 eggs were below the limit of detection (10^1.5^ TCID_50_/mL) even in E1 eggs (data not shown). We then used an rRT-PCR assay to quantify the viral RNA levels in the inoculum and allantoic fluid samples (*10*). Viral RNA decreased steadily over the 3 passages in eggs (Figure 3, panel B). We also inoculated chicken embryo fibroblasts with USA-WA1; no infectious virus was produced from the cells (Figure 3, panel A). These results clearly demonstrate that embryonated chicken eggs are not a susceptible substrate for SARS-CoV-2 replication. Collectively, the data show that substrates commonly used to culture influenza A and B viruses are not susceptible to SARS-CoV-2 infection.

### Replication of SARS-CoV-2 in Polio and Enterovirus Substrates

**Figure 4:**
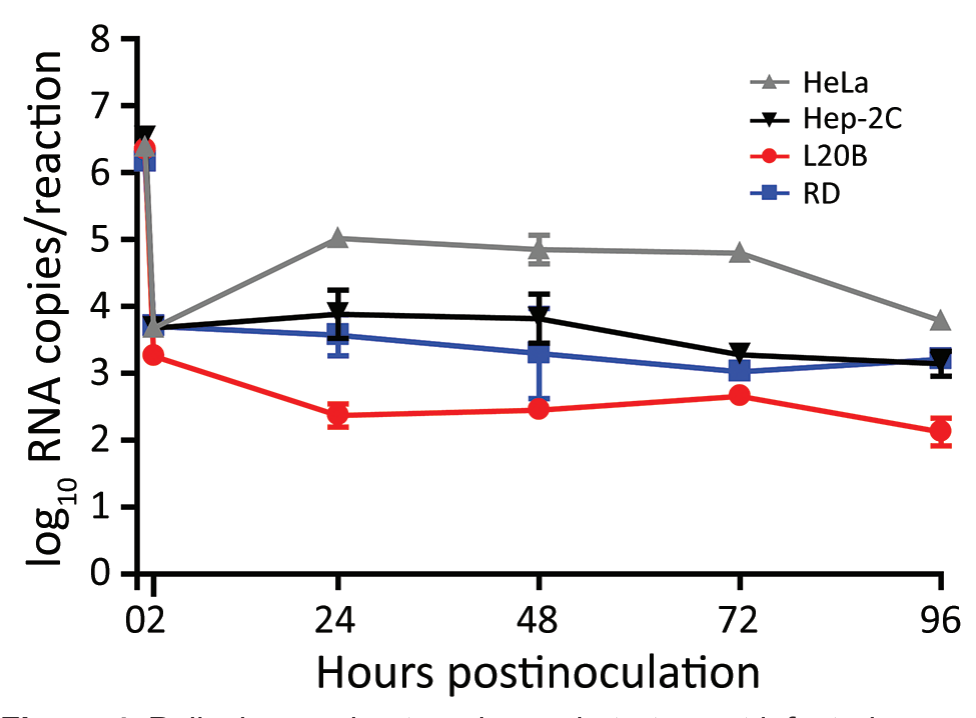
Poliovirus and enterovirus substrates not infected by severe acute respiratory syndrome coronavirus 2 (SARS-CoV-2) in study of susceptibility to SARS-CoV-2 of cell lines and substrates used to diagnose and isolate influenza and other viruses. Total viral RNA levels determined by real-time reverse transcription PCR (standard curve generated by synthetic RNA) from RNA extracted from cell lines inoculated with USA-WA1 at MOI 0.1 in 6-well plates. Data points at 1 h represented by RNA from the inoculum; >2 h time points from RNA extracted from cell lysates. Data are mean of n = 3 +SD.

Stool specimens from patients potentially infected with polio or enteroviruses are used to inoculate appropriate cell lines for surveillance. Because SARS-CoV-2 virus can infect multiple organs and tissues and its presence in stool specimens has been reported (*14**–**20*), it is important to determine if cell lines commonly used for polio and enterovirus culture could inadvertently propagate SARS-CoV-2. Therefore, we inoculated RD, HeLa, Hep-2C, and L20B cells with USA-WA1 at a multiplicity of infection (MOI) of 0.1 and incubated for 2 hours after which we removed the inoculum and washed the cells 3 times to remove residual virus. We observed no cytopathic effect over a 4-day period and SARS-CoV-2 was not detectable in supernatant collected at 1–4 dpi (data not shown). This result was confirmed by rRT-PCR of cell lysate, which revealed that the total viral RNA levels decreased relative to the inoculum, indicating that virus did not efficiently initiate RNA transcription or replication (Figure 4). These results indicate that cell substrates regularly used for polio and enterovirus cultures are not susceptible to SARS-CoV-2 infection when cultured under standard conditions.

### Replication of SARS-CoV-2 with Spike D614G Substitution

**Figure 5:**
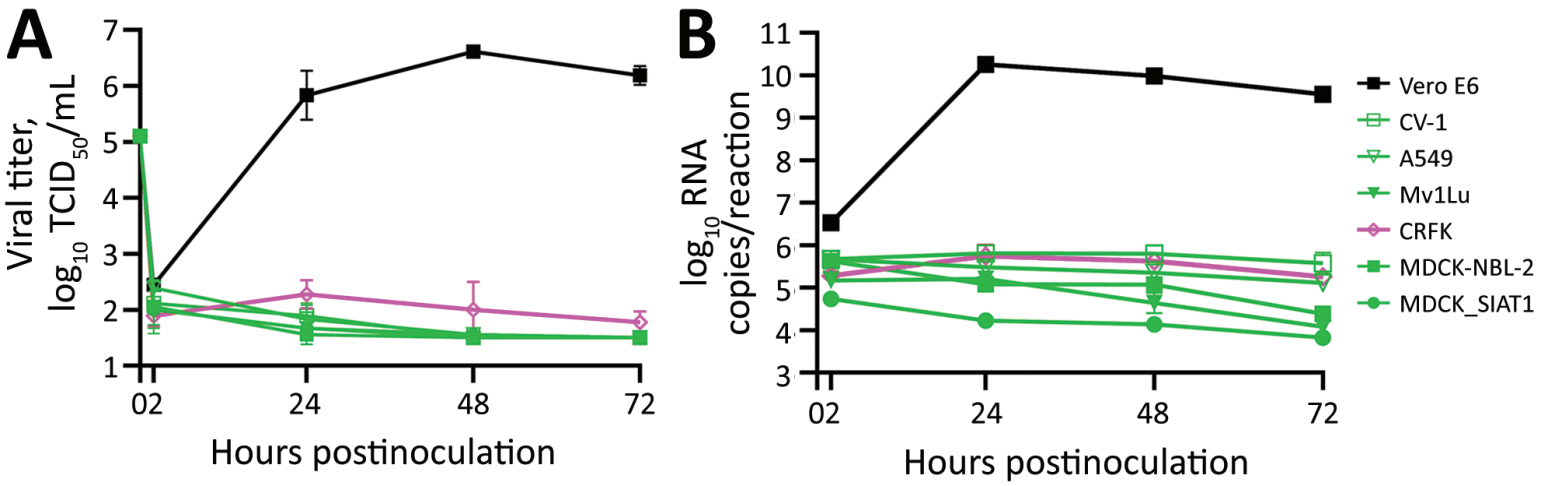
Infection of severe acute respiratory syndrome coronavirus 2 (SARS-CoV-2) with spike G614 in study of susceptibility to SARS-CoV-2 of cell lines and substrates used to diagnose and isolate influenza and other viruses. Vero E6, CV-1, A549, Mv1Lu, CRFK, MDCK-NBL-2, and MDCK-SIAT1 cell lines inoculated with MA/VPT1 at 5 × 10^5^ TCID_50_/well in 12-well plates (MOI 1 to ≈5 depending on cell line). A) Supernatants collected at indicated times and used to determine viral replication kinetics by TCID_50_. B) Total viral RNA levels extracted from cells inoculated for the indicated times as determined by real-time reverse transcription PCR. Data are mean of n = 3 +SD. TCID_50_, median tissue culture infectious dose.

During this study, we noticed that the proportion of naturally circulating virus containing a D614G substitution in the spike protein was rapidly increasing. The USA-WA1 strain is an early isolate that expresses spike with D614. To confirm that the cell susceptibility data obtained using this virus were valid with recent strains, a subset of representative cell lines were inoculated with a high titer (5 × 10^5^ TCID_50_/well) of SARS-CoV-2/Massachusetts/VPT1/2020 (MA/VPT1), which encodes a spike with G614. In selecting cell lines for the subset, we included Vero E6 cells as a cell line that should support replication of MA/VPT1 given our previous findings with USA-WA1 (Figure 3, panel A). Indeed, Vero E6 cells supported similar replication kinetics for MA/VPT1 and USA-WA1 (Figure 5, panel A). Even with a 10-fold higher inoculum of MA/VPT1 than previously used for USA-WA1 tests (5 × 10^4^ TCID_50_/well), CV-1, A549, Mv1Lu, MDCK-NBL-2, and MDCK-SIAT1, cell lines were not susceptible to this SARS-CoV-2 strain encoding spike G614. CRFK cells inoculated with MA/VPT1 generated virus titers slightly above the limit of detection at 1 dpi, after which titers decreased (Figure 5, panel A). We further confirmed viral titers by rRT-PCR. Consistent with the virus titer data, inoculated CRFK cells had a 5-fold increase of viral RNA at 1 dpi compared to 2 hpi, but the RNA levels decreased over the next 2 days. In contrast, CV-1, A549, Mv1Lu, MDCK-NBL-2, and MDCK-SIAT1 cells did not shown any noticeable increase of viral RNA levels during the time course of this study (Figure 5, panel B). All 7 cell lines in this subset demonstrated very similar viral replication kinetics for both MA/VPT1 and USA-WA1 virus strains (Figures 2–5), indicating that the currently dominant virus strains with spike G614 likely have the same cell susceptibility profile as earlier strains encoding spike D614.

### ACE2 as a Critical Determinant in Susceptibility and Species Specificity

**Figure 6:**
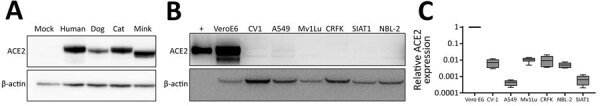
ACE2 differentially expressed across cell lines in study of susceptibility to severe acute respiratory syndrome coronavirus 2 of cell lines and substrates used to diagnose and isolate influenza and other viruses. A) Mock transfected 293T cells or 293T cells transfected with plasmids expressing human, dog, cat, or mink ACE2 immunoblotted for ACE2 protein expression. B) Whole-cell lysate from uninoculated Vero E6, CV-1, A549, Mv1Lu, CRFK, MDCK-NBL-2, and MDCK-SIAT1 cell lines immunoblotted for endogenous ACE2 expression. Recombinant human ACE2 used as a positive control for detecting human ACE2. C) Relative ACE2 expression determined by real-time quantitative PCR. Data are mean of n = 6 +SD. Boxes are 1 SD away from the mean, and whiskers indicate the minimum and maximum. ACE, angiotensin-converting enzyme 2.

Coronavirus spike-host receptor interactions play the major role in species specificity (*21*). SARS-CoV-2 uses hACE2 as the host cell receptor (*22*). Multiple species, including humans, monkeys, cats, minks, ferrets, hamsters, and dogs, have been infected by SARS-CoV-2 in experimental and natural settings (*23*–*28*). To further investigate the mechanism of susceptibility or resistance and gain insight into SARS-CoV-2 species specificity, we analyzed the ACE2 expression levels in various cell lines. Multiple ACE2 antibodies were screened to identify a polyclonal antibody that reacts with transiently overexpressed ACE2 in humans, dogs, cats, and minks (Figure 6, panel A). Using this antibody, we determined by immunoblot that endogenous ACE2 levels were very high in Vero E6 cells derived from African green monkey kidneys but extremely low in the other African green monkey kidney cell line, CV-1, which could explain the drastic difference in infectivity between these 2 cell lines (Figure 6, panel B). Canine ACE2 protein was not detectable in MDCK cells, which surely plays a role in their resistance to SARS-CoV-2 infection. Similarly, feline CRFK, mink Mv1Lu, and human A549 cells had very low or undetectable endogenous ACE2 expression (Figure 6, panel B). The low protein levels of ACE2 in those cells coincided with low mRNA levels determined by rRT-PCR (Figure 6, panel C).

**Figure 7:**
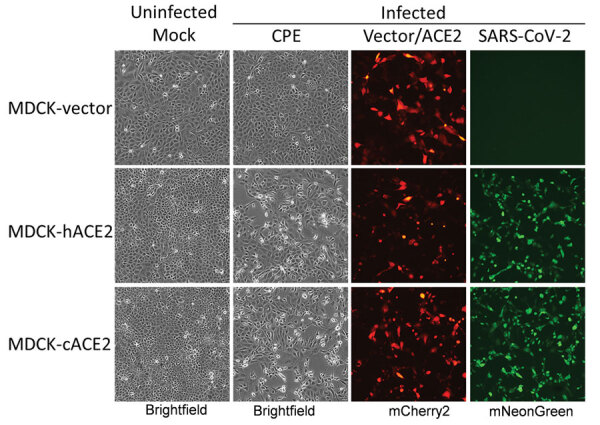
Overexpression of canine ACE2 in MDCK cells in study of susceptibility to severe acute respiratory syndrome coronavirus 2 of cell lines and substrates used to diagnose and isolate influenza and other viruses. Cells inoculated with icSARS-CoV-2-mNG reporter virus. Representative images at 1 dpi are shown (original magnification ×10). ACE, angiotensin-converting enzyme 2.

**Figure 8:**
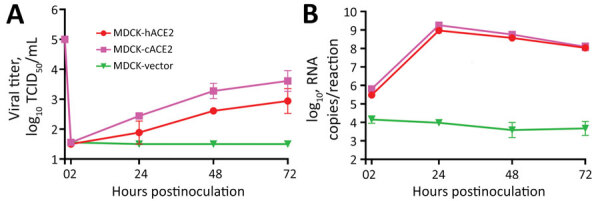
MDCK-vector, MDCK-hACE2, and MDCK-cACE2 cells inoculated with USA-WA1 at 5 × 10^5^ TCID_50_/well in 12-well plates in study of susceptibility to severe acute respiratory syndrome coronavirus 2 of cell lines and substrates used to diagnose and isolate influenza and other viruses. Supernatants collected at the indicated times. A) Viral titers determined by TCID_50_ assay; B) total viral RNA determined using real-time reverse transcription PCR (standard curve generated by synthetic RNA). Data for both panels are mean of n = 3 +SD. ACE, angiotensin-converting enzyme 2; cACE2, canine ACE2; hACE2, human ACE2; TCID_50_, median tissue culture infectious dose.

Since MDCK cells are the most important cell line for isolating and propagating influenza viruses and dogs have been infected with SARS-CoV-2, we selected cACE2 for additional analysis. To better understand resistance of MDCK cells to SARS-CoV-2, we transfected constructs coexpressing hACE2 or cACE2 proteins under a cytomegalovirus promoter and mCherry2 protein through an IRES element into MDCK-SIAT1 cells. MDCK cells expressing hACE2 (MDCK-hACE2) or cACE2 (MDCK-cACE2) as determined by mCherry2 expression were efficiently infected by icSARS-CoV-2-mNG (Figure 7). We also transfected MDCK cells with an empty vector plasmid that expresses mCherry2 via the IRES element but does not encode an ACE2 protein (MDCK-vector) as a control. Like wild-type MDCK cells, the MDCK-vector control cells were not susceptible to SARS-CoV-2 (Figure 7). We further confirmed these results by infecting MDCK-hACE2 and MDCK-cACE2 cells with the wild-type virus USA-WA1 and assaying viral replication kinetics. Viral infectious titers and viral RNA levels were elevated in MDCK cells overexpressing either hACE2 or cACE2 relative to MDCK-vector cells (Figure 8, panels A, B).

**Figure 9:**
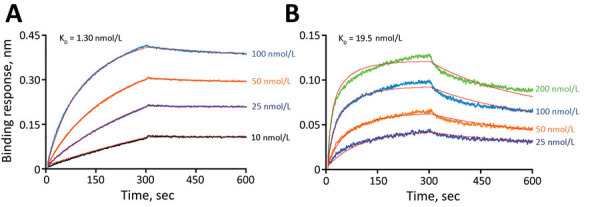
Canine ACE2 affinity to severe acute respiratory syndrome coronavirus 2 (SARS-CoV-2) spike protein compared with that for human ACE2 in study of susceptibility to SARS-CoV-2 of cell lines and substrates used to diagnose and isolate influenza and other viruses. Biolayer interferometry assay used to determine K_D_, the equilibrium dissociation constant of human ACE2 or canine ACE2 protein with SARS-CoV-2 spike protein.

**Figure 10:**
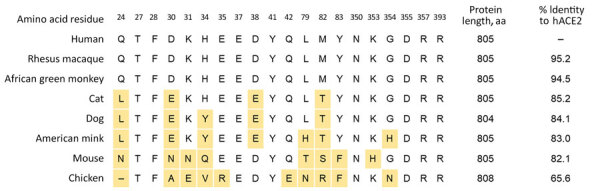
Aligned ACE2 protein sequences from human, rhesus macaque, African green monkey, cat, dog, American mink, mouse, and chicken cells in study of susceptibility to severe acute respiratory syndrome coronavirus 2 (SARS-CoV-2) of cell lines and substrates used to diagnose and isolate influenza and other viruses. Residues involved in interaction with SARS-CoV-2 spike protein (*41**–**44*) shown using hACE2 numbering; yellow indicates residues varying from hACE2. Dash indicates gap in alignment. Percentage identity to hACE2 across the entire protein is shown. ACE, angiotensin-converting enzyme 2; cACE2, canine ACE2; hACE2, human ACE2.

These results indicate that MDCK cell resistance to SARS-CoV-2 occurs at the virus entry step. Once bound, the genome is released, transcribed, translated, replicated, and packaged into particles that efficiently bud from infected cells. However, overexpression of ACE2 in MDCK cells could result in greater ACE2 expression than in most natural cell lines. Therefore, even if cACE2 does not bind the spike protein as efficiently as hACE2, overexpression could facilitate entry of SARS-CoV-2 into MDCK-cACE2 cells. To determine if cACE2-binding affinity to SARS-CoV-2 spike was an additional factor preventing infection of MDCK cells, we conducted biolayer interferometry assays to compare the binding affinity of spike with cACE2 and hACE2. We identified that the SARS-CoV-2 spike bound to cACE2 (equilibrium dissociation constant [K_D_] = 19.5 nmol/L) 15-fold less efficiently than to hACE2 (K_D_ = 1.30 nmol/L) (Figure 9). The reduced binding affinity to cACE2 is likely a result of the sequence differences between the hACE and cACE2 in regions directly involved in spike binding (Figure 10). Therefore, both low expression of cACE2 by MDCK cells and low binding affinity of cACE2 to SARS-CoV-2 spike contribute to the resistance of MDCK cells to SARS-CoV-2.

## Discussion 

In this study, we determined the SARS-CoV-2 susceptibility of >30 cell lines and derivatives and embryonated chicken eggs. Findings from our study corroborate and complement those from other susceptibility studies published in recent months (*29*,*30*), including that MDCK cells and embryonated eggs do not support productive SARS-CoV-2 infection (*30*). In addition, our infectious virus titration assay data further showed that SARS-CoV-2 loses infectivity rapidly in cells and eggs, whereas the viral RNA levels decreased slowly. In addition, most circulating strains contain the D614G substitution in the spike protein, which could affect binding, entry, and species specificity; viruses with this change were not tested in previous studies. Herein, we showed that the spike D614G substitution does not alter susceptibility of the cell lines tested including those with low levels of human (A549), nonhuman primate (CV-1), mink (Mv1Lu), cat (CRFK), or dog (MDCK) ACE2. In the future, even in the unlikely event that other spike substitutions render the binding of spike to cACE2 stronger (Figure 9), the low expression level of cACE2 in MDCK cells (Figure 6) still poses a high barrier for SARS-CoV-2 to overcome. Therefore, 2 independent studies together illustrate that MDCK cells and commonly used derivatives are not susceptible to SARS-CoV-2 and can be safely used for isolating and propagating influenza viruses and producing vaccines. In addition, chicken eggs, which are used to manufacture most influenza virus vaccines, do not support replication of SARS-CoV-2.

We expanded our examination to other clinically relevant cell lines used in diagnosis and isolation of a wide array of human viruses, particularly respiratory viruses (Table 1). Although many of those cells were tested with SARS-CoV-1 virus previously (*29*,*31*–*40*), it is worth noting that cell susceptibility conclusions derived from SARS-CoV-1 studies do not always apply to SARS-CoV-2. For example, we and others previously showed that Mv1Lu cells supported a moderate level of SARS-CoV-1 virus replication (*35*,*38*), but they are not susceptible to SARS-CoV-2 replication, as demonstrated in this study. This finding could be justified by the difference in ACE2 binding positions between SARS-CoV-1 and SARS-CoV-2 (*41*–*44*). Considering that mink ACE2 is only 83% identical to human ACE2 (Figure 10), some of the different ACE2 residues may have more adverse effect on SARS-CoV-2 entry than on SARS-CoV-1 entry. This idea does not necessarily contradict recent reports of SARS-CoV-2 infections among minks on farms (*24*,*45*–*48*); ACE2 expression is relatively low in Mv1Lu cells (Figure 6) but likely higher in various epithelial cells in vivo, enabling productive infection in minks in spite of a weaker spike-receptor interaction.

Overall, our study provides useful information on multiple cell lines and chicken eggs regarding their susceptibility to SARS-CoV-2. Of note, from a biosafety standpoint, humans can be co-infected with multiple pathogens. Specimens collected for testing and culture of other viruses may contain SARS-CoV-2; these data should help laboratories avoid inadvertent propagation. The data on canine ACE2 shed light on the relationship between SARS-CoV-2 susceptibility and ACE2 receptor affinity (species specificity) and expression level, suggesting that even ACE2 proteins with several substitutions at key residues that contact SARS-CoV-2 spike protein can still serve as functional receptors when expressed at high levels.
